# Maternal and Congenital Toxoplasmosis: Diagnosis and Treatment Recommendations of a French Multidisciplinary Working Group

**DOI:** 10.3390/pathogens8010024

**Published:** 2019-02-18

**Authors:** François Peyron, Coralie L’ollivier, Laurent Mandelbrot, Martine Wallon, Renaud Piarroux, François Kieffer, Eve Hadjadj, Luc Paris, Patricia Garcia –Meric

**Affiliations:** 1 Civil hospices of Lyon. Department of Parasitology and Mycology, Croix Rousse Hospital, 69317 Lyon, France; martine.wallon@chu-lyon.fr; 2 Assistance Publique-Hôpitaux de Marseille, Department of Parasitology, Timone Hospital, 13005 Marseille, France; Coralie.LOLLIVIER@ap-hm.fr; 3 Assistance Publique-Hôpitaux de Paris, Department of Obstetrics and Gynecology, Louis Mourier Hospital, Colombes, Université Paris-Diderot, Paris, Inserm UMR1137 IAME: Infection, 75013 Paris, France; laurent.mandelbrot@aphp.fr; 4 Sorbonne Université, INSERM, Institut Pierre-Louis d’Epidémiologie et de Santé Publique, AP-HP, Hôpital Pitié-Salpêtrière, 75651 Paris, France; renaud.piarroux@aphp.fr; 5 Assistance Publique-Hôpitaux de Paris, Department of Neonatology, Trousseau Hospital, 75012 Paris, France; francois.kieffer@aphp.fr; 6 Assistance Publique-Hôpitaux de Marseille, Ophtalmology Consultations, Sainte Marguerite Hospital, 13274 Marseille, France; Eve.HADJADJ@ap-hm.fr; 7 Assistance Publique-Hôpitaux de Paris, Department of Parasitology-Mycology, Pitié-Salpêtrière/Charles Foix University Hospital, 75651 Paris, France; luc.paris@aphp.fr; 8 Assistance Publique-Hôpitaux de Marseille, Department of Neonatal Medicine; Hospital of the Conception, 13005 Marseille, France; Patricia.GARCIA@ap-hm.fr

**Keywords:** congenital toxoplasmosis, guidelines, treatment of fetal infections, follow-up of congenital infection

## Abstract

Women infected with toxoplasmosis during pregnancy do not present symptoms in most cases, but the consequences of the congenital infection may be severe for the unborn child. Fetal damage can range from asymptomatic to severe neurological alterations to retinal lesions prone to potential flare up and relapses lifelong. Despite the possible severity of outcome, congenital toxoplasmosis (CT) is a neglected disease. There is no consensus regarding screening during pregnancy, prenatal/postnatal treatment or short or medium term follow-up. Since 1992, France has offered systematic serological testing to non-immune pregnant women, monthly until delivery. Any maternal infection is thus detected; moreover, diagnosis of congenital infection can be made at birth and follow-up can be provided. “Guidelines” drawn up by a multidisciplinary group are presented here, concerning treatment, before and after birth. The recommendations are based on the regular analysis of the literature and the results of the working group. The evaluation of the recommendations takes into account the robustness of the recommendation and the quality of the evidence.

## 1. Introduction

Toxoplasmosis is a cosmopolitan disease caused by an intracellular parasite, *Toxoplasma gondii*, which affects one third of the world population. Infection occurs via the digestive tract after ingestion of contaminated food, water or soil. Primary infection induces protective immunity of the host, observed by the presence of specific antibodies. Infection is most often subclinical, however severe and sometimes fatal symptoms can occur in immunocompromised patients and in the fetus. Congenital toxoplasmosis (CT) is a global public health issue, in view of its potential severity. In pregnant women, since infection is usually asymptomatic, it can be detected only by serological testing. Preventive and diagnostic measures for this disease in pregnant women vary widely between countries [1]. Although prenatal diagnosis of CT is available, and despite the fact that fetal lesions can be severe, there is no international framework for monitoring the disease and it is a neglected disease in most countries. At birth, CT is often ignored, even though it can lead to ocular lesions and neurological disorders. Many caregivers are not aware of the specifics of CT, and are therefore ill at ease to inform future parents. Screening during pregnancy is not performed in most countries [2], and it has been criticized in view of the low incidence of the disease, the cost and the lack of formal proof of treatment efficacy in this context. A few countries, including France and Austria, provide serological screening to pregnant women. In France, screening for toxoplasmosis in pregnancy has been mandatory since 1992, with serological testing at the first-trimester prenatal visit, with monthly serologic follow-up until delivery of pregnant women identified as “not protected against toxoplasmosis”.

To date, guidelines for management of maternal and congenital toxoplasma infections have yet to be published, despite more than 30 years of experience, and common practices in the leading centers in France [1]. The goal of this article is to propose recommendations in order to both prevent CT in case of maternal infection and, if CT does occur, to provide necessary care before and after birth. A French multidisciplinary working group drafted recommendations, addressing the following questions:

How should women be informed about toxoplasmosis?
How to manage pregnant women infected with toxoplasmosis?
How to manage neonates born to mothers with toxoplasmosis infection during pregnancy?
How to treat and follow infants with congenital toxoplasmosis?

## 2. Methodology

### Expert Panel Composition

The working group was composed of experts from the different fields involved in CT: an obstetrician, two pediatricians, five parasitologists and an ophthalmologist. The group reviewed the literature published in peer-reviewed journals and findings from the databases of Paris, Lyon and Marseille concerning over 5000 maternal seroconversions and the regular follow-up of 1000 patients with CT, ranging from newborns to patients in their 40s. The working group convenes once per year in order to update the recommendations using data from recent literature and the experience of its members. Criteria used to measure the strength of recommendations and the levels of evidence are presented in annex 1 [3] and indicated in bold type, in brackets in the text. In case of weak or insufficient evidence, the guidelines were based on expert opinion. A consensual document was elaborated and then validated by independent experts (see acknowledgements).

## 3. Natural History of Congenital Toxoplasmosis

### 3.1. The Parasite

The life cycle of *T. gondii* involves definitive hosts (cats and other felines) in which the parasite reproduces sexually. Resistant infective forms (oocysts) are passed in feces, thus contaminating soil, fruits, vegetables or water. Animals, domesticated meat animals in particular (especially pigs, lambs, less frequently cows), ingest oocysts incidentally. In these new hosts (intermediate hosts), the ingested parasite transforms into a rapidly multiplying form in the digestive tract, which can go through the intestinal wall and disseminate, affecting other organs. After this phase of parasitemia, lasting around 10 days, under the pressure of the immune system, the parasites morph into the dormant cyst form, persisting in particular in the brain and striated muscle tissue where they maintain the protective immune status of the host [4].

The same phases of the life cycle occur in humans. During the phase of parasitemia, *T. gondii* can pass through the placenta; thus, it is crucial to treat as early as possible. Only one species of *Toxoplasma* exists, but it possesses great genetic diversity. It thrives all over the world and has different levels of virulence [5]. In Europe, isolates are, for the most part, of type 2. In North America, type 2 coexists with type 3, but other types have been described in wild and domestic fauna. In South America, many genotypes have been described, associated with greater virulence. This genetic diversity shows that the clinical and epidemiological profile of the disease is not uniform and that the impact of CT on public health must be evaluated country by country.

### 3.2. Epidemiology

In pregnant women, toxoplasmosis is acquired through the consumption of undercooked meat infected with cysts, or through oocysts infecting vegetables or water in nature. In Brazil, serologic testing has suggested the possibility of infection through drinking water [6]. This last route of infection certainly deserves more attention with respect to prevention and clinical consequences. A decrease in prevalence has been recorded in most developed countries. In France, prevalence in pregnant women was 80% in the 1960s; it fell to 31% in 2016 [7]. This trend can be explained by various factors: more widespread consumption of frozen meat, modern mass-produced meat production, better hygiene, urbanization, etc.

### 3.3. Pathophysiology and Outcome of Congenital Toxoplasmosis

During the period of parasitemia following a primary infection, the parasite may pass through the placenta. The more mature the placenta, the easier the passage. The risk of fetal infection therefore increases with gestational age. At 6, 18 and 30 weeks’ gestation, the risk of fetal infection is 2.2%, 23% and 56%, respectively (Table 1).

In contrast, the risk of severe CT is inversely proportional to gestational age. Fetal infection in early pregnancy may lead to adverse outcomes including spontaneous abortion or brain damage. On the contrary, fetal infections occurring in late pregnancy are frequent, but usually subclinical. Thus, the gestational age at the moment of maternal infection is crucial to evaluate fetal risk; antenatal diagnosis and treatment are based on this information. Manifestations of CT are polymorphic, ranging from fetal death, to severe neurological and ocular damage, to absence of any clinical signs. Management of this last situation is difficult if healthcare providers have no information about maternal infection and the newborn is clinically healthy; there would be no reason to screen the infant for CT. Yet, ocular lesions may develop later on in life. Of more than 2000 infections of pregnant women followed prospectively and treated, 79% of newborns were not affected by CT [9] and birth defects occurred in less than 1% of cases. The outcome of congenital toxoplasmosis must be considered over the long term. Quality of life was assessed using the PGWBI (psychological general well-being index) questionnaire [10] on a cohort of 102 adults with CT, most of whom had been treated before and after birth and received regular follow-ups; the global score results were similar to that of the control population [11]. In the same cohort, 58.8% of patients presented with ocular lesions, but quality of vision—derived from the VF14 questionnaire [12]—was high enough that the patients gave global scores of 97.3 out of 100. Thirteen subjects presented with low visual acuity. This situation is associated with neurological lesions, strabismus or retinal damage near the fovea. Immunity may decrease around the age of 30, and systematic follow-up of CT in adults has allowed us to identify pregnancy as the period most at-risk for reactivation of chorioretinitis. Moreover, women with congenital toxoplasmosis must be reminded to respect the rules of prevention against toxoplasmosis during pregnancy [13]. Information regarding the evolution of CT after the age of 40 has not yet been collected. Its role in neuro-cognitive disorders, as reported by several observational studies on toxoplasmosis positive patients, would be noteworthy [14,15].

## 4. How Should Women Be Informed about Toxoplasmosis?

Only 30% of women with primary toxoplasmosis infection present clinical signs [16], and these are non-specific. The most effective method to detect toxoplasmosis is serological screening.

According to the guidelines of the French High Authority for Health [17], screening for toxoplasma immunoglobulins(Ig) IgG and IgM should be offered during preconception care and at the first prenatal visit, as early as possible in the first trimester of pregnancy.

If the maternal serology is negative, women must be informed about measures to prevent infection with *T. gondii*, and sequential serological follow-up should be performed. In case of the appearance of anti-toxoplasmosis IgM and specific IgG, the gestational age at the moment of maternal infection is easy to evaluate and the risk and severity of fetal damage can be estimated [8]. When IgM appears without specific IgG, further testing is required to diagnose recent infection because of the possibility of non-specific IgM. Other assays can be used to confirm the specificity of these antibodies and the appearance of IgG in some instances.

If the initial maternal serology is positive for IgG and negative for IgM, this indicates infection before pregnancy.

In case of the presence of both IgG and IgM, further testing is required to estimate the timing of infection with respect to the date of conception. This is difficult to establish, especially if the first test is performed late in pregnancy. The presence of IgM, which may persist for years, is not absolute evidence of recent toxoplasmosis. Algorithms of interpretation, using different assays to titre immunoglobulin, IgG avidity and sequential serological testing should be used to determine the date of maternal infection [18]. Collaboration with a laboratory specializing in toxoplasmosis is necessary in this situation.

## 5. How to Manage Pregnant Women Infected with Toxoplasmosis

The care offered for an infection requires different treatments whose indications would eventually depend on the amniocentesis results when performed. These results would provide the diagnosis of a potential fetal infection and ultrasound would detect fetal malformation.

### 5.1. Treatments

There are two possible protocols. Spiramycin is generally used for prophylaxis with the objective of preventing fetal infection. The other option is the combination of pyrimethamine and sulphonamides (PS), which should be avoided before 14 weeks of gestation. In France, PS is generally reserved for CT diagnosed by positive amniocentesis or third-trimester maternal infection in the absence of amniocentesis, with the objective of reducing the severity of fetal damage. These two protocols are active only on rapidly multiplying parasitic forms and are inactive on cysts.

Spiramycin (Protocol 1)

- Dose: 3 million units (1 g), 3 times per day
- Tolerance is good, although adverse effects may occur, mostly minor gastrointestinal intolerance. Taking the medication during a meal improves tolerance.
- Efficacy: Spiramycin accumulates in the placenta, which may prevent fetal infection. Publications report that this treatment, if initiated early enough after maternal infection, may reduce the risk of fetal infection (reviewed in [19]) even if no randomized controlled study has been reported showing the efficacy of this treatment in the prevention of fetal infection or in the reduction of sequelae [20].
- Spiramycin is not effective on established congenital toxoplasmosis [19]

Pyrimethamine–sulfamides combinations (Protocol 2)

Two different regimens are used:

Pyrimethamine1 tablet of 50 mg dailySulfadiazine3 tablets of 500 mg twice dailyFolinic acid2 capsules of 25 mg per week

*OR*

Sulfadoxine–pyrimethamine combination * Folinic acidCapsules equivalent to Fansidar^®^ (500 mg/25 mg) must be prepared: 2 capsules per week 2 capsules of 25 mg per week* Sulfadoxine and pyrimethamine can be ordered from the Inresa laboratory (1, rue Jean Monnet F-68870 Bartenheim).

In case of treatment by pyrimethamine–sulfamide, supplementation with folinic acid is required (folic acid is not effective). Women should be instructed to drink at least 2 L per 24 h and to alkalinize the urine (for instance, by consuming citrus-based products [21]).

A history of G6PD deficiency is considered as a contra-indication, although no clear relation between anemia and G6PD deficiency was found in pregnant women receiving sulfadoxine–pyrimethamine for intermittent preventive treatment of malaria [22]. In case of such deficiency, the patient should be addressed to a reference center.

Surveillance: The blood cell count (BCC) must be followed up every 1 to 2 weeks. In case of neutropenia (neutrophils count < 1500/mm^3^), stop treatment and continue folinic acid, perform a new BCC test 1 week later and reinitiate treatment according to results. (Opinion of the working group.)

Tolerance: Good tolerance has been reported, in general [16,23,24]. Three studies report no case of hematologic toxicity under pyrimethamine and sulfadiazine with folinic acid supplemention. In a cohort of 1007 pregnant women receiving the same medications, but at a different dose, 5 experienced adverse effects, 4 of which resolved spontaneously, and 1 case of allergic reaction to sulfadiazine–pyrimethamine was reported with a favorable evolution [16].

Efficacy in CT: Observational studies have reported the efficacy of this treatment on fetal infection, especially if the treatment is initiated in the 4 weeks following maternal infection [16,23,25,26]. The severity of fetal damage is more difficult to evaluate as the consequences are extremely variable. It is not possible to place all clinical manifestations of congenital toxoplasmosis on the same scale, as they range from debilitating neurological damage, to small retinal lesions with no impact on vision, to cerebral calcifications with no clinical manifestations. Moreover, ocular lesions can appear later in life [27], thus it is important that children with CT be offered ophthalmological follow-up until adulthood. It is difficult to grasp the impact of CT on quality of life and its global burden, with regards to disability. Observational studies have reported attenuation of clinical signs following antenatal treatment [28,29,30]. The comparison between two cohorts of children infected by the same type of parasite (type 2) showed a rate of hydrocephaly of 31% without antenatal treatment and a rate of 0.8% if the mother was treated [9]. PS is used in some countries beyond the first trimester as prophylaxis in case of maternal infection. To date, only one randomized clinical trial has been reported [24]. This study suggested that PS was more effective than spiramycine in preventing CT in cases of maternal toxoplasmosis seroconversion, however the difference did not attain statistical significance due to a small sample size. Additionally, the incidence of prenatal ultrasound cerebral signs was significantly lower in the group receiving PS than S.

### 5.2. Amniocentesis

Prenatal diagnosis should be offered with an amniocentesis to perform a PCR assay for toxoplasmosis DNA in the amniotic fluid [31]. This procedure should not be performed before 18 weeks of gestation (WG) nor less than 4 weeks after the estimated date of maternal infection, in order to reduce the risk of false negatives due to delayed transplacental passage of the parasite [18]. The sensitivity and the specificity of current PCR testing are 92% and 100%, respectively [32]. The positive and negative likelihood ratios of the test vary according to the incidence, thus depend on gestational age at the moment of infection and help to estimate with precision the risk of fetal infection (Table 1). The risk of procedure-related fetal loss (or preterm delivery in more advanced gestation) is estimated in recent studies to be less than 0.1% [33]. An amniocentesis is useful, since appropriate care can be provided during pregnancy and for the neonate in case of a positive result, and the parents can be reassured in the case of a negative result. There is no indication for medical termination of pregnancy on the basis of positive amniocentesis alone (cf. infra).

### 5.3. Recommendations of the Working Group

#### 5.3.1. First-Trimester Infections

The risk of transplacental passage is low.

We recommend prescribing protocol 1 as soon as possible, until the result of prenatal diagnosis (Appendix A1A) or until delivery of amniocentesis is forgone and the fetal ultrasound normal.

In case of preconceptional infection, it is admitted that the fetus is not at risk of congenital toxoplasmosis. For periconceptional infection (1 month before or after conception) the risk is very low and the decision of whether to perform an amniocentesis should be discussed with the mother, in view of the benefit–risk ratio. One must wait until 18 WG to perform an amniocentesis.

In case of a negative amniocentesis result, continue protocol 1 until delivery to avoid possible transplacental passage of the parasite, which may still occur after the amniocentesis (Appendix A1C). In case of significant intolerance, the benefit–risk ratio is in favor of therapeutic abstention. (Opinion of the working group.)

In case of a positive amniocentesis result, prescribe protocol 2 (Appendix A1B). In case of intolerance to the treatment, pyrimethamine–azithromycin (250 mg per day) combination used for the treatment of ocular toxoplasmosis [34] may be considered. (Opinion of the working group.)

#### 5.3.2. Second-Trimester Infections

Protocol 1 or protocol 2 [24,35] should be started as soon as possible.

Amniocentesis is recommended, in order to adapt therapy and follow-up (Table 1) (Appendix A1B).

In case of a negative amniocentesis result, continue or switch to protocol 1 until delivery to avoid possible transplacental passage of the parasite, which may still occur after the amniocentesis (Appendix A1B).

In case of a positive amniocentesis result, continue or prescribe protocol 2 immediately (Appendix A1C).

#### 5.3.3. Third-Trimester Infections

Because of the high risk of transmission, protocol 2 should be started as soon as possible.

An amniocentesis should be considered, since in case of a positive PCR result, prompt prenatal and neonatal therapy can be offered (Appendix A1C). In late pregnancy, the safety of the procedure should be carefully evaluated by the prenatal diagnosis specialist.

In case of a negative amniocentesis result, switch to protocol 1 until delivery to avoid possible transplacental passage of the parasite, which may still occur after the amniocentesis. In case of significant intolerance, the benefit–risk ratio is in favor of stopping prophylactic therapy. (Opinion of the working group.)

In case of a positive amniocentesis result, continue protocol 2 (Appendix A1B).

If an amniocentesis is not performed, protocol 2 should be prescribed (Appendix A1C), as the fetal risk is high. This situation must be clearly explained to the parents.

#### 5.3.4. Prenatal Follow-Up

Ultrasound follow-up should be performed monthly, since abnormalities may appear several weeks or months after infection (Appendix A2A). In case of a positive amniocentesis, the follow-up may be intensified to every 2 weeks (Appendix A1B). This evaluation should be performed by a sonographer specialized in prenatal diagnosis and CT (opinion of the working group). Fetal brain MRI is not recommended routinely; however, it may be performed in cases where the interpretation of ultrasound imaging is difficult.

#### 5.3.5. Obstetrical Management

Termination of pregnancy should be considered only in case of ultrasound anomalies associated with poor outcome, essentially hydrocephalus. Other anomalies, in particular isolated cerebral parenchymal lesions, have not been found to be related to neurological impairment [36], and must be evaluated by experts in CT before considering termination of pregnancy. In French law, termination for severe fetal anomalies can be performed, without a gestational age limit, only upon approval by the Multidisciplinary Centre of Prenatal Diagnosis after written maternal request. If the pregnancy is continued, protocol 2 must be continued as well (Appendix A1B).

There is no evidence that performing prompt delivery by caesarean or induction of labor, in order to spare the fetus from further transplacental risk, has any benefit for the prevention of transmission [37], thus this practice does not appear justified (Appendix A2C).

## 6. How to Manage Neonates Born to Mothers with Toxoplasmosis Infection during Pregnancy

In cases of proven maternal seroconversion, neonatal clinical examination should be performed. In addition:

- Fundus examination and transfontanellar ultrasound should be performed in case of positive prenatal diagnosis. Lumbar puncture is not necessary in most cases [4].
- When an amniocentesis was not performed, as well as in cases of a negative amniotic fluid PCR, in order not to misdiagnose congenitally infected neonates, neonatal work-up must be performed (opinion of the working group). The first test can be performed using cord blood or peripheral blood, usually sampled on day 2 or 3. The sera must be sent to a specialist laboratory possessing the necessary assay equipment to test for specific IgM and IgA in neonates, and to perform a comparative mother–infant Western blot [4]. IgG that can cross the placenta are not markers of CT. The presence of IgM and/or IgA must be systematically confirmed with blood drawn from the infant after three days of life. Serological tests may differ, but their performances are generally good [28].
- Supplementary tests may be available in some centers, including PCR for parasite detection on the placenta, cord blood or in amniotic fluid at delivery. The reliability of the results is variable.

The decision to treat is made in light of the results of the prenatal and neonatal examinations. Section 6.1, Section 6.2, Section 6.3 describe three situations that may arise.

### 6.1. Negative Neonatal Results and, If Performed, Negative Antenatal Results

This is the most frequent situation in France, accounting for 79% of cases.

Serological follow-up is required to confirm the absence of CT. Testing should be performed at 1 month and every 2 months thereafter, showing a decreasing antibody titer, until complete disappearance of anti-toxoplasma IgG (of maternal origin), confirming the absence of CT [38], which usually occurs before the age of 1 year. Complete disappearance of anti-toxoplasma antibodies must be obtained before stopping serological follow-up (Appendix A1A). The tests must always be performed in the same laboratory so as to obtain coherent kinetic results.

If the diagnosis of CT is proven by serological evolution (synthesis of IgG, IgM and/or IgA), a treatment and surveillance must be initiated according to the protocols mentioned above (cf. infra).

In case of maternal infection in late pregnancy, testing at birth may be falsely negative. Do not start the presumptive treatment, as this may hide diagnostic markers, but repeat serological testing more frequently. Sera should be taken at 2 weeks, 1 month, months and then every 2 months until results show complete disappearance of anti-toxoplasmosis IgG (opinion of the working group).

In case of the appearance of CT markers, start treatment (cf. infra). Positive serological results at the age of one year are proof of CT. At this stage of the disease, it is likely that the infant carries only cysts that are resistant (inaccessible) to treatment.

### 6.2. Positive Antenatal and/or Neonatal Testing: Confirmed Congenital Toxoplasmosis

Treatment should be started as soon as possible

Treat continuously for 12 months with one of the three following protocols (Table 2):

Adverse effects may be more severe with the combination of sulfadoxine–pyrimethamine than with pyrimethamine–sulfadiazine and are more likely to occur during the first 2 months of treatment. However, the administration of the combination of sulfadoxine–pyrimethamine (once per week) is simpler, thereby increasing the chances of adherence to this long-term treatment.

The pyrimethamine, sulfadiazine and sulfadoxine capsules must be prepared by a hospital pharmacy according to the weight of the infant.

In the case of active ocular lesions, ophthalmologists may prescribe corticosteroids.

#### Surveillance during the Treatment

Recommendations of the working group are as follows:

– Make sure there is no G6PD deficiency (a condition more frequently observed in Africa and Asia).
– Check BCC at D0 and D15, and thereafter once per month. In case of neutropenia (neutrophil count < 800/mm^3^), stop anti-toxoplasmosis treatment and continue administration of folinic acid. Check CBC 2 weeks later, and reinitiate treatment when the neutrophil count reaches > 800/mm^3^.
– In the case of severe cutaneous manifestations, stop treatment immediately and definitively.
– Clinical, ophthalmological and serological surveillance should be offered every 3 months.

Transient diasappearance of anti-toxoplasma IgG may occur under treatment—do not take into account [39]

In cases of toxoplasmosis with severe or moderate manifestations, early initiation of treatment has been associated with improvement [40]. Treatment is also recommended in asymptomatic newborns in order to prevent delayed occurrence of ocular lesions [19], even if the efficacy of this protocol has not been demonstrated by randomized studies (opinion of the working group).

Tolerance of treatment is generally good. Among 65 children treated for 1 year, 13.8% presented at least 1 benign clinical adverse effect and 9.2% experienced a hematological adverse event, possibly related to treatment [41]. A systematic review on a total of 929 children treated with pyrimethamine and sulfadiazine or sulfadoxine did not report any serious adverse events. The most frequent adverse effects were hematological, dermatological and/or gastrointestinal, which were mild or moderate [42]. As the treatment is well tolerated and is effective on overt toxoplasmosis and the parents wish the best chances for the child, the working group recommends treating all children with congenital as quickly as possible. The indication and duration of therapy may be discussed in case of asymptomatic toxoplasmosis, but the treatment is imperative in the presence of neurological and ophthalmological signs (Appendix A1C).

An infant treated for CT may be breastfed and receive vaccinations (opinion of the working group).

### 6.3. Discordance between Neonatal and Prenatal Test Results

In some cases, PCR on amniotic fluid and perinatal work up yield discordant results. As mentioned above, when PCR results on amniotic fluid are positive, the diagnosis of congenital toxoplasmosis is nearly certain, whereas in cases of negative amniocentesis results, there remains a risk of false negatives, even with high-quality PCR techniques [39,40], or of delayed transmission occurring after the amniocentesis. The pre-test probability of fetal infection varies according to the gestational age at the time of maternal infection. When PCR is positive and specific, IgM and IgA in newborns are negative and the probability of CT is very high. Comparative mother–infant Western blot, a test exploring cellular immunity [43], and/or sequential serological testing can be performed (opinion of the working group). When PCR is negative but IgM and IgA positive, complementary testing and follow-up are required.

## 7. How Should Infants with Congenital Toxoplasmosis Be Observed?

Transient negative serology is frequently observed in infants under therapy. If the infant is diagnosed with congenital toxoplasmosis, this should not be questioned and regular monitoring should be continued [39] (Appendix A1C). By the end of the treatment a serological rebound is very frequently observed [44]. This rebound does not signify that treatment should be reinitiated (Appendix A1C). Breast feeding is not contra-indicated and immunization programs should be carried out according to local recommendations (opinion of the working group).

Clinical and ophthalmological surveillance should be continued (opinion of the working group):

- every 3 months during the second year of life;
- every 6 months during the third year of life;
- once per year lifelong.

The duration of the follow-up is subject to discussion. The only concern is the unpredictable occurrence or recurrence of chorioretinitis, especially during the first 10 years of life [27]. From the age of 5 years, even if the child is supposedly able to describe the appearance of visual disorders, we suggest systematic clinical and ophthalmic monitoring [45]. This is the only way to have unbiased information on the evolution of the ocular risk of this disease. The data collected from follow-up provides information on the evolution of the disease that can be used to reassure and educate pregnant women concerning the evolution of the disease. At present, the group advocates for clinical and ophthalmological follow-up for life. Furthermore, 98% adult patients of our CT cohort requested to participate in follow-up [45].

### New Ophthalmological Events Occurring during Follow-Up

Recurrences of choriorentinitis can occur unpredictably throughout childhood and adolescence [46]. Pregnancy can provoke recurrence [47].

Treat only if active lesions are detected during fundus exam, especially if the area affected is close to the fovea centralis or the optic nerve (Appendix A1C). In this case, reinitiate treatment (protocol 1 or 2) for 3 months and check regularly the healing of the lesions (Appendix A1C).

A recent meta-analysis reported that the combination of trimethoprim–sulfamethoxazole could be an alternative treatment [48].

The efficacy of corticosteroids has yet to be proven. They are recommended only if the visualization of the fundus is severely hindered. In cases of foveal lesion in the better eye of the patient, clearing of the vitreous should be done as soon as possible. There is no place for steroids without antiparasitic coverage, the worst being intravitreal steroids without sufficient antiparasitic therapy, which will result in severe retinal necrosis. Rare cases like optic nerve head affection might be treated in specialized centers (opinion of the working group).

The subjects in our cohort, in the vast majority of cases, live normal lives [11]. The only restriction is that they must avoid ocular trauma (combat sports) that could reactivate existing chorioretinitis lesions (opinion of the working group).

## 8. Conclusions

Our purpose was to propose effective management of pregnant women who have a suspected or confirmed infection, and of newborns at risk of CT. Our recommendations are limited by the lack of randomized controlled trials and the large heterogeneity of published retrospective cohort studies. Our guidelines were based on the analysis of published studies and the population-based data from systematic monthly follow-ups of pregnant women in France since 1992, which allowed centers to recruit non-biased patients and screen all at-risk newborns at birth. The performances of serological tests were quite satisfactory with the assays available today, and prenatal diagnosis methods (amniocentesis, fetal ultrasound) were safe and accurate. There is reasonably good evidence of the efficacy of therapy in cases of positive results. Under these conditions of care, the absence of fetal infection was observed in 79% of cases of maternal infection and the number of terminations of pregnancy for fetal damage was less than 1%. The different treatments used were generally well-tolerated by the mother, fetus and child, but require information and careful surveillance. There is a need for further study on the efficacy of various treatment regimens. The evaluation of the quality of life and visual function in a cohort of adults with CT showed a good long-term prognosis of this condition. However, there is concern regarding a possible impact of toxoplasmosis on behavioral disorders. Whatever the decision of public health authorities, pregnant women should be informed about toxoplasmosis and its prevention measures. Furthermore, clinicians should consider the diagnosis of toxoplasmosis in pregnant women with clinical symptoms and signs, such as cervical lymphadenopathy, and consider CT in fetuses with ultrasound abnormalities compatible with “TORCH” infections. The manageable evolution of CT observed in our setting may be linked to the weak virulence of the type II strain most commonly observed in France, but also to the prompt care that is offered in case of maternal infection; therefore, one must keep in mind that this might not be the case for other countries. Research should be performed to assess the burden of CT in each country, the cost of care of the disease before birth should be estimated and studies should be performed to validate recommendations that would appropriate for each country.

## Figures and Tables

**Table 1:** Evolution of the probability of fetal infection with respect to gestational age at the moment of maternal infection and the result by PCR (according to [8]).

Gestational Age at the Time of Maternal Infection			
	6 WG	18 WG	30 WG
Pre-test probability of congenital toxoplasmosis			
(%)	2.2	23.0	56
Positive likelihood ratio	79 (29->1000)	69 (34->1000)	43 (20->1000)
Probability of fœtal infection (%)	64.0 (39.0–100)	95.4 (91.0–100)	98.2 (96.2–100)
Negative likelikhood ratio	0.43 (0.10–0.78)	0.37 (0.25–0.48)	0.23 (0.12–0.36)
Probability of fetal infection (%)	1.0 (0.2–1.7)	10.0 (7.0–12.5)	22.6 (13.2–31.4)

WG: weeks of gestation.

**Table 2:** Post natal treatment for infants with congenital toxoplasmosis.

The Three Protocols	
(1)	
Pyrimethamine +	1 mg/kg/day for 2 months, then 0.5 mg/kg/day
Sulfadiazine	50 mg /kg twice daily
Folinic acid	1 capsule of 25 mg twice per week, starting on the same day as the treatment
(2)	
Sulfadoxine + pyrimethamine	17.5 mg/kg once per week 0.875 mg/kg once per week
Folinic acid	1 capsule of 25 mg twice per week, starting on the same day as the treatment
(3)	
Pyrimethamine and sulfadiazine as in Protocol 1 for the first 2 months, to test for tolerance, then sulfadoxine–pyrimethamine as in Protocol 2 for the remaining 10 months (see above for dosage)	

## Appendix A. Classification of the grade of recommendation and the evidence used to produce these guidelines [3].

**Grade of Recommendation**	**Clarity of Risk/Benefit**	**Quality of Supporting Evidence**	**Implications**
**1A.** Strong recommendation, high quality evidence	Benefits clearly outweigh risk and burdens, or vice versa.	Consistent evidence from well-performed randomized, controlled trials or overwhelming evidence of some other form. Further research is unlikely to change our confidence in the estimate of benefit and risk.	Strong recommendations, can apply to most patients in most circumstances without reservation. Clinicians should follow a strong recommendation unless a clear and compelling rationale for an alternative approach is present.
**1B.** Strong recommendation, moderate quality evidence	Benefits clearly outweigh risk and burdens, or vice versa.	Evidence from randomized, controlled trials with important limitations (inconsistent results, methodologic flaws, indirector imprecise), or very strong evidence of some otherresearch design. Further research (if performed) is likely to have an impact on our confidence in the estimate of benefit and risk and may change the estimate.	Strong recommendation and applies to most patients. Clinicians should follow a strong recommendation unless a clear and compelling rationale for an alternative approach is present.
**1C.** Strong recommendation, low quality evidence	Benefits appear to outweigh risk and burdens, or vice versa.	Evidence from observational studies, unsystematic clinical experience or from randomized, controlled trials with serious flaws. Any estimate of effect is uncertain.	Strong recommendation, and applies to most patients. Some of the evidence base supporting the recommendation is, however, of low quality.
**2A.** Weak recommendation, high quality evidence	Benefits closely balanced with risks and burdens.	Consistent evidence from well-performed randomized, controlled trials or overwhelming evidence of some other form. Further research is unlikely to change our confidence in the estimate of benefit and risk.	Weak recommendation, best action may differ depending on circumstances or patients or societal values.
**2B.** Weak recommendation, moderate quality evidence	Benefits closely balanced with risks and burdens, some uncertainly in the estimates of benefits, risks and burdens.	Evidence from randomized, controlled trials with important limitations (inconsistent results, methodologic flaws, indirect or imprecise), or very strong evidence of some other research design. Further research (if performed) is likely to have an impact on our confidence in the estimate of benefit and risk and may change the estimate.	Weak recommendation, alternative approaches likely to be better for some patients under some circumstances.
**2C.** Weak recommendation, low quality evidence	Uncertainty in the estimates of benefits, risks and burdens; benefits may be closely balanced with risks and burdens.	Evidence from observational studies, unsystematic clinical experience, or from randomized, controlled trials with serious flaws. Any estimate of effect is uncertain	
